# An Interactive Framework of Cross-Lingual NLU for In-Vehicle Dialogue

**DOI:** 10.3390/s23208501

**Published:** 2023-10-16

**Authors:** Xinlu Li, Liangkuan Fang, Lexuan Zhang, Pei Cao

**Affiliations:** School of Artificial Intelligence and Big Data, Hefei University, Hefei 230061, China; xinlu.li@hfuu.edu.cn (X.L.); fanglk@stu.hfuu.edu.cn (L.F.); zhanglx@stu.hfuu.edu.cn (L.Z.)

**Keywords:** interactive framework, in-vehicle dialogue, contrastive learning, attention mechanism, cross-lingual

## Abstract

As globalization accelerates, the linguistic diversity and semantic complexity of in-vehicle communication is increasing. In order to meet the needs of different language speakers, this paper proposes an interactive attention-based contrastive learning framework (IABCL) for the field of in-vehicle dialogue, aiming to effectively enhance cross-lingual natural language understanding (NLU). The proposed framework aims to address the challenges of cross-lingual interaction in in-vehicle dialogue systems and provide an effective solution. IABCL is based on a contrastive learning and attention mechanism. First, contrastive learning is applied in the encoder stage. Positive and negative samples are used to allow the model to learn different linguistic expressions of similar meanings. Its main role is to improve the cross-lingual learning ability of the model. Second, the attention mechanism is applied in the decoder stage. By articulating slots and intents with each other, it allows the model to learn the relationship between the two, thus improving the ability of natural language understanding in languages of the same language family. In addition, this paper constructed a multilingual in-vehicle dialogue (MIvD) dataset for experimental evaluation to demonstrate the effectiveness and accuracy of the IABCL framework in cross-lingual dialogue. With the framework studied in this paper, IABCL improves by 2.42% in intent, 1.43% in slot, and 2.67% in overall when compared with the latest model.

## 1. Introduction

The intelligent development of in-vehicle dialogue systems is one of the main goals to improve the functional convenience and driving safety of automobile cockpits [1,2,3,4,5]. Natural language understanding (NLU) is crucial for the implementation of voice control functions in in-vehicle dialogue systems, which mainly consists of two tasks: intent detection and slot filling. Intent detection means that when the user inputs a command to the dialogue systems, the computer determines whether the question is to turn on the air conditioner, listen to music, query the navigation location, etc. Slot filling is to determine the specific content of the intent based on the intent detection. For example, if the user inputs a navigation command to ‘go to the airport’, the dialogue system first determines that the intent of this sentence is to navigate, and the specific navigation location is the airport.

Since 2019, with the development of pretrained models in the field of natural language processing (NLP) and the improvement of representation capabilities, researchers have proposed a variety of joint-training frameworks [6,7,8,9,10,11,12,13] based on pretrained models to improve the ability of cross-lingual NLU. These frameworks have been applied in the industrial domain [6], cross-lingual interactive chatbots [7], cross-lingual e-banking domain [8], restaurant customer service domain [9], airline ticket purchasing customer service domain [10,11,12,13], and so on. These studies [6,7,8,9,10,11,12,13] show that the representation capability of pretrained models and the application of cross-lingual transfer learning techniques can play an important role in multilingual NLU in different domains.

To summarize, researchers have carried out applied research on cross-lingual NLU in many fields, but so far, there is no research on cross-lingual NLU tasks of a dialogue system in the field of in-vehicle dialogue. According to market research, the existing cross-lingual NLU in the vehicle field mainly adopts the rule-based method [14]. However, with the addition of some new knowledge and new terms, the rule-based method needs to spend a lot of manpower and material resources to adjust. In addition, there are currently private datasets in the field of in-vehicle dialogue, so it is necessary to build a dataset for an in-vehicle dialogue system, which can be shared and used by other researchers to promote the development of an in-vehicle dialogue system.

This paper focuses on the in-vehicle domain, attaining multilingual NLU and providing a set of datasets for multilingual in-vehicle dialogue. The contributions of this paper are mainly as follows:

(1) This paper provided a set of datasets of NLU tasks of an in-vehicle dialogue system under cross-lingual scenarios, including Chinese, Arabic, Japanese, and English. The intent and slots of the datasets are annotated according to the annotation specification of the currently published MultiATIS++ [10] dataset.
(2) It proposed a cross-lingual interactive framework for in-vehicle human–computer dialog systems. The framework employs an end-to-end architecture for joint modeling, using pretrained models and incorporating contrastive learning to communicate the relationship between intent recognition and slot filling tasks through interactive attention mechanisms in downstream tasks.
(3) It validated an IABCL framework on self-built MIvD datasets and public datasets. To the best of our knowledge, this is the first time to evaluate the model work on the joint task of intent detection and slot filling in in-vehicle dialogue systems. The experimental results show that the attention-based interactive framework achieves remarkable results in cross-lingual intent detection and slot filling.

## 2. Related Work on Cross-Lingual NLU

### 2.1. In-Vehicle Dialogue NLU

Under the impetus of economic globalization, the number of automobile users has been growing gradually and the languages they use are different, so it is an urgent problem for NLU in the automobile field to correctly identify the intent detection and slot filling of the user’s expressions in cross-lingual environments.

This paper investigates cross-lingual NLU tasks in other domains. For example, in the industrial domain, in order to perform multilingual intent classification under low resources, Khalil et al. [6] constructed an industrial intent dataset and explored different methods for multilingual intent detection in industrial environments. The presented methods [6] verified that the source language can be well migrated to the target language based on the machine translation model and the multilingual pretraining model. In 2020, in the cross-lingual interactive chatbot domain, in order to better utilize cross-lingual transfer learning techniques to improve the accuracy of chatbots in multilingual prediction, especially for the COVID-19-related problem, Arora et al. [7] proposed a joint model that is structured for simultaneous intent detection and slot filling, and compared the effectiveness of different pretrained models in the cross-lingual transfer learning task. In addition, the authors provide a multilingual dataset, M-CID, containing English, Spanish, French, and German. In 2021, Daniela et al. [8] combined machine translation with a pretrained model for an intent detection task in the e-banking domain and obtained excellent results, and this article also contributed a new data resource called MIN DS-14. In the restaurant customer service domain, in order to reduce the cost of cross-lingual NLU, Liu et al. [9] proposed an attention-informed mixed-language training method. This mixed language training method uses a very small number of task-related parallel word pairs to generate sentences in a hybrid language. It outperforms the then zero-sample adaptation methods in dialogue state tracking and natural language understanding, and is less costly. In the field of ticketing customer service in the aircraft aviation domain, Xu et al. [10] proposed a new end-to-end model in order to address slot alignment and recognition in cross-lingual migration, which outperforms the traditional simple tag mapping method using fast-align on most languages, and the authors also released a new multilingual NLU dataset, MultiATIS++. Jack et al. [11], in the field of aircraft aviation, described a new multilingual NLU task that simultaneously translates input text, outputs text with slots, categorizes intents, recognizes the input language, and improves on the MultiATIS++ dataset using the multilingual BART model. In the same area, in 2020, Qin et al. [12] proposed a new approach to improve the language neutrality of Transformer-based language models (e.g., mBERT) by switching data through multilingual codes for fine-tuning downstream NLU tasks. Compared with machine translation, multilingual code switching allows for multilingual text characterization at a finer granularity, such as the sentence, phrase, or word level, while the authors released a new manually annotated tweet dataset for intent detection and slot filling in English and Haitian. In 2022, to address the problem of sequential alignment between multilingual transitions in the field of aircraft aviation, Qin et al. [13] proposed a contrastive learning framework. The framework achieves excellent performance on ticketing customer service in the aviation domain and successfully brings the representations of cross-lingual similar sentences closer together.

### 2.2. Cross-Lingual Transfer Learning

In the field of single-language dialogue system, NLU based on deep learning has achieved remarkable results [15]. However, in the field of multilingualism, especially in low-resource languages, the lack of a large amount of training data makes the model of intent detection and slot filling not better aligned between different languages, so its recognition effect in the cross-lingual domain is not significant. In recent years, some researchers have adopted the concept of transfer learning to train NLU models in resource-rich languages, such as English and Chinese, and adopted cross-lingual transfer learning strategies to apply them to other low-resource languages [16].

Cross-lingual transfer learning is a machine learning technique that allows knowledge learned in one language to be applied to another. The basic idea is to transfer the knowledge of the language model of one or more source languages into the language model of the target language [17]. There are two main approaches to cross-lingual transfer learning: one based on machine translation and the other based on a multilanguage model. The approach based on machine translation is to translate resource-rich data into a resource-poor target language and then train the model of the target language. For example, in 2010, Lefevre et al. [18] proposed a method to complete the intent detection task in the target language through machine translation given the source language and its semantic annotations. In 2017, Adams et al. [19] proposed a method using cross-lingual word vectors to solve the natural language processing problem of low-resource languages. It uses bilingual dictionaries to link monolingual corpora of two different languages to learn word embeddings in both languages in a common vector space, thereby helping to solve problems such as data scarcity and lack of labeled data in low-resource languages, improving the performance of natural language processing tasks. Andrew et al. [20] proposed a method of MOM (mixed orthographic mapping) to overcome the spelling differences between English and Japanese and realize the application of cross-lingual transfer learning in Japanese-named entity recognition. Another approach based on multilingual models is usually taking a pretrained model with some modifications and fine-tuning. In 2020, Qin et al. [12] proposed a multilingual code-switching data enhancement framework to fine-tune mBERT to align the representation of the source language and multiple target languages, improving the performance of cross-lingual NLU tasks. Qin et al. [13] proposed to use contrast learning to narrow the representation of cross-lingual similar sentences in mBERT to solve the word order alignment problem between multilanguage code transitions and achieved outstanding performance.

### 2.3. Joint Learning Frameworks for Intent Detection and Slot Filling

Joint learning of intent detection and slot filling is the mainstream model training method in the field of NLU at present. Parallel architecture is the earliest joint training method, which treats the two tasks as parallel subtasks instead of serial ones when processing intent detection and slot filling. Li et al. [21] constructed a parallel architecture of intent detection and slot filling. The architecture adopted bidirectional LSTM as the embedding layer, introduced attention-based classifiers for intent detection, and modeled slot filling with a conditional random field (CRF). Schuster et al. [22] used a self-attentional classifier as intent detection in the decoding layer, filled the CRF modeling slot, and compared some multilanguage models in the encoder. The advantage of a parallel architecture is that both intent detection and slot filling can be performed simultaneously, thus improving the efficiency and performance of the model. However, the disadvantage is that it ignores the strong correlation between the two tasks, and cannot supervise each other in the process of transfer learning, so the overall accuracy of the model is affected.

Aiming at the bidirectional interaction between intent and slot, Zhang et al. [23] proposed a dual-model architecture in 2016 to consider the cross-impact between intent and slot by using two related bidirectional LSTMS. Wang et al. [24] proposed to share parameters of RNN (Joint ID and SF) to learn the correlation between intent and slot. Compared with parallel architecture, the results of both intent detection and slot filling can be significantly improved with the same number of parameters. Liu et al. [25] then introduced a shared encoder–decoder framework with an attention mechanism, and introduced attention mechanisms into RNN models that provide additional information for intent classification and slot filling. Compared with shared RNN parameters, the bidirectional interaction for intent detection and slot filling has improved the results. In 2019, Chen et al. [26] proposed a BERT-based joint intent classification and slot filling model, which uses a universal large-scale language to represent the model BERT compared with the RNN model, reducing the training time. In 2021, Zhou et al. [27] introduced a new parallel interaction network (PIN) in which the interaction between intent detection (ID) and slot filling (SF) tasks is divided into two phases: an implicit interaction phase and an explicit interaction phase. In the implicit interaction phase, ID and SF tasks transmit information through the bidirectional recurrent neural network, and in the explicit interaction phase, ID and SF tasks fuse information through a collaborative mechanism. This interaction achieved the most advanced results at the time. In 2021, Qin et al. [28] used a co-interactive module to establish a bidirectional connection between two tasks. This module allows slots and intents to pay attention to each other’s information, to better capture the cross-influence between the two tasks, and this interaction is evaluated and improved on ATIS and SNIPS. In 2022, Qin et al. [12] added the correlation between the learning intents and slots of three contrast learning modules by introducing the contrastive learning framework. The results of cross-lingual intent detection and slot filling have been significantly increased on the MultiATIS++ dataset. It is therefore promising to consider the interaction between these two tasks.

To sum up, strategies based on multilanguage models are commonly used in the field of cross-lingual transfer learning. The advantages of such strategies are that they can reduce training costs, improve efficiency, and enhance generalization ability. In addition, the bidirectional interactive framework is often used in the joint learning framework, which can make the model capture more shared knowledge between tasks, thus improving the performance of both tasks. Second, the bidirectional interactive framework helps to improve the interpretation ability of the model, making it easier to analyze the effects between slots and intents.

Therefore, this paper will adopt the strategy of a multilanguage model and propose an attention-based bidirectional interactive framework based on a bidirectional interactive framework to realize cross-lingual intent detection and slot filling in the field of in-vehicle dialogue.

## 3. Modeling Framework

**Figure 1:**
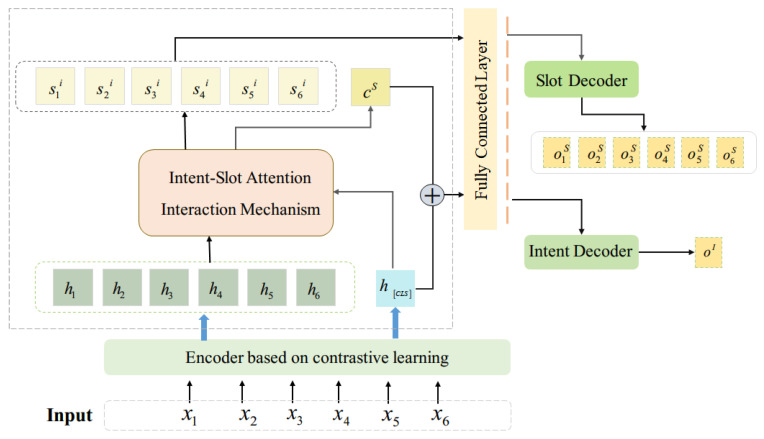
Interactive attention model based on contrastive learning.

The proposed overall framework shown in Figure 1 has two parts: the encoder based on contrastive learning and the decoder based on interactive attention. The encoder is mainly responsible for converting text characters into embedded vectors for deep learning and projecting the vectors onto a high-dimensional plane. Contrastive learning can help models learn discrimination feature representations. By comparing the source statement with the positive and negative samples, the model learns the feature representation that can accurately distinguish the positive and negative samples. The positive and negative samples in this paper are multilingual text representations with the same semantics and different semantics as the source text, respectively. Therefore, the encoder based on contrast learning uses high-dimensional embedded vectors to carry out standardized multilanguage learning through self-supervised means, which enables the encoder to integrate more language information in the coding stage and increase the cross-lingual ability of the model. The decoder based on interactive attention uses the characteristics of coding to generalize intent detection and slot filling. In this process, these features are extracted and used for learning, while interactive attention is used for two-way interaction between intent detection and slot filling.

### 3.1. Contrastive-Learning-Based Encoder

An encoder converts an input sequence into a fixed-length embedding, such as a piece of text. This embedding is called a “context vector” or “encode vector”. The code vector contains information about the input sequence and acts as the input to the decoder to help it generate the output sequence.

Contrastive learning (CL) is a machine learning method that learns differences by comparing positive and negative samples. Contrastive learning helps NLP models learn meaningful representations of text data. The model learns to capture semantic and contextual information by contrasting positive and negative samples, enabling better understanding of word meanings, sentence structures, and overall language semantics. It has been widely used in computer vision, natural language processing, and other fields, and performs well [29].

**Figure 2:**
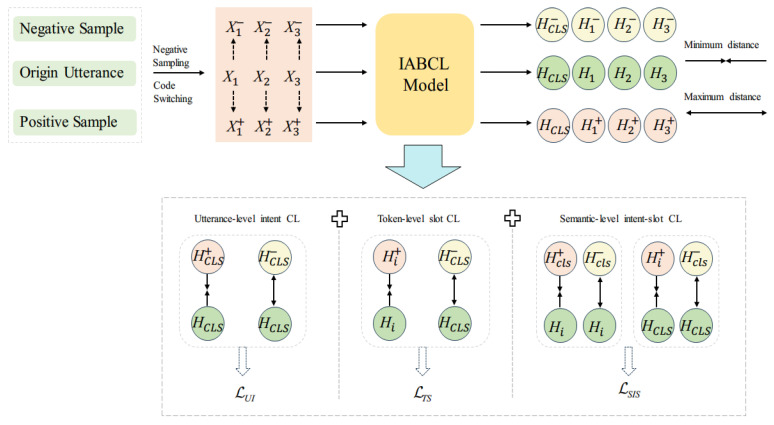
Contrastive-learning-based encoder.

As shown in Figure 2, the model mainly consists of a multilanguage pretraining model and a contrastive learning module. The multilanguage pretraining model is pretrained based on various texts, which can be mBERT, XLM-R, and other models. Contrastive learning modules include a sentence-level intent CL module, word-level slot CL module, and semantic level intent-slot CL module. The sentence-level intent CL module is used to distinguish the intents of texts in different languages with the same semantics; the word-level slot CL module is used to distinguish the slots of words in different languages with the same semantics; and semantic-level intent-slot CL module is used to learn the correlation between intent slots.

The contrastive learning adopted in this paper is to take the new sentence expression obtained from the translation of the words in the source sentence as the positive sample and the expression with different meanings as the negative sample.

#### 3.1.1. Positive Sample

A positive sample is a multilingual statement with the same semantics. First, given a source statement $X^{}=\left\{x_{1}^{},x_{2}^{},x_{3}^{},\ldots ,x_{n}^{}\right\}$, a positive sample $X^{+}=\left\{x_{1}^{+},x_{2}^{+},x_{3}^{+},\ldots ,x_{n}^{+}\right\}$ is generated by a bilingual dictionary. Specifically, a positive sample is obtained by randomly selecting each word $x_{1}$ in X and replacing $x_{1}$ with a translation improved by a bilingual dictionary. For example, given the English source statement watch football match, the positive sample is *看 (zh/watch) サッカー (ja/football) Спички (es/match)*.

#### 3.1.2. Negative Sample

A negative sample is very different from a positive sample. A source statement generally generates only one positive sample statement, but a negative sample is a collection of statements. The negative sample set is generated in such a way that the coding samples of previous batches will be reused, thus eliminating unnecessary negative coding processes.

The whole encoder process: First of all, the source utterance X carries out multilanguage code switch data to obtain $X^{+}\;$ and $X^{-}\;$, and then input to the mBERT encoder to obtain the representation H, $H^{+}$, $H^{-}$.

#### 3.1.3. Loss Function of Contrastive Learning

Three contrastive learning modules consisting of positive samples $X^{+}\;$, negative samples $X^{-}\;$, and source utterances X as inputs are the sentence-level intent CL module, the word-level slot *CL* module, and the semantic-level intent-slot CL module.

1. Sentence-level intent *CL* module for aligning sentence representations across languages for intent detection.

Intent detection is a sentence-level classification task. In the cross-linguistic domain, aligning sentence representations is an important measure for the zero-shot cross-linguistic intent detection task. Sentence alignment can explicitly encourage models to align similar sentence representations into the same space across languages for intent detection. Here is the sentence-level intent contrastive learning loss calculated for a sentence:

(1) $$L_{UI}=-\log\frac{s(h_{CLS},h_{CLS}^{+})}{s(h_{CLS},h_{CLS}^{+})+s(h_{CLS},h_{CLS}^{-})}$$

2. Word-level slot *CL* module for cross-lingual alignment of slot representations for slot filling.

Since slot filling is a word-level task, cross-lingual alignment of sentence representations is used to help the model consider the alignment of slot filling for fine-grained cross-lingual transfer. Here is the word-level slot contrastive learning loss for the *i*-th word:

(2) $$L_{TS}^{i}=-\sum_{i=1}^{n}\log\frac{s(h_{i},h_{i}^{+})}{s(h_{i},h_{i}^{+})+s(h_{i},h_{i}^{-})}/n$$

3. Semantic-level intent-slot *CL* module for aligning representations between slots and intents.

When slot and intent belong to the same object, they are usually highly semantically related. Therefore, this paper argues that intents and their slots in sentences constitute a positive correlation, while the corresponding slots in other sentences constitute a negative correlation. Therefore, this paper further introduces semantic-level intent-slot *CL* loss to model the semantic interaction between slots and intents, which may further improve the cross-linguistic transfer between them:

(3) $$L_{SIS1}=-\sum_{i=1}^{n}\log\frac{s(h_{CLS}^{+},h_{i})}{s(h_{CLS}^{+},h_{i}^{})+\sum_{k=0}^{K-1}s(h_{CLS}^{-},h_{i,k}^{})}/n$$

(4) $$L_{SIS2}=-\sum_{i=1}^{n}\log\frac{s(h_{CLS},h_{i}^{+})}{s(h_{CLS},h_{i}^{+})+\sum_{k=0}^{K-1}s(h_{CLS},h_{i,k}^{-})}/n$$

(5) $$L_{SIS}=L_{SIS1}+L_{SIS2}$$

where $L_{SIS1}$ and $L_{SIS2}$ are the contrastive learning loss between the positive samples of the intent vector and slot vector and the source utterance, respectively, and s(a,b) represents the dot product of a and b.

### 3.2. Interactive-Learning-Based Decoder

In neural networks, decoders play a crucial role in various tasks that involve generating output from abstract representations or latent features. Decoders are often paired with encoders, which are responsible for converting input data into a compressed or abstract representation. The decoder’s role is to transform these representations back into the desired output format.

The decoder stage adds an interactively learned multitask attention head to the fully connected layer. Because of the high correlation between the intent detection and slot filling tasks, the hidden state of the slot filling task is shared to the intent detection task through the attention mechanism in the decoder phase, and the hidden state of the intent detection task acts on the slot filling as well. Intention detection task and slot filling task learn together and promote each other.

The intent detection attention head and slot filling attention head are computed based on the output sequence of the pretrained model by calculating the attention computation score, which is combined with the fully connected layer for different tasks to compute the final state.

1. Intent detection task attention head:

(6) $$\alpha^{I}=\frac{exp\left(h_{CLS}\right)}{exp\left(h_{CLS}\right)+\sum_{i=1}^{n}\exp\left(h_{i}\right)}$$

2. Slot filling task attention header:

(7) $$\alpha_{i}^{S}=\frac{exp\left(h_{i}\right)}{exp\left(h_{CLS}\right)+\sum_{i=1}^{n}\exp\left(h_{i}\right)}$$

The intent detection task is to put $h_{CLS}$ into the classification layer to find labels:

(8) $$o^{I}=softmax\left(\;W^{I}h_{CLS}^{out}+\alpha_{i}^{S}h_{i}^{out}+b^{I}\right)$$

There, W and b are the matrix of the parameter to be trained. In this paper, we jointly fine-tune all the parameters of mBERT and W by maximizing the logarithmic probability of correct labeling.

For the sequence labeling task, this paper feeds the final hidden state of the model to the softmax layer to classify the slots. In this paper, the hidden state corresponding to the first sublabel is used as input to classify words:

(9) $$o^{S}=softmax\left(W^{S}\;h_{i}^{out}+\alpha^{I}h_{CLS}^{out}+b^{S}\right),i=1,2,\ldots n$$

### 3.3. Multitask Learning

The loss function is to calculate the difference between the forward computed result and the true value of each iteration of the neural network, to guide the next step of training in the right direction. Its input is the network prediction and the real target value, and then calculates a distance value, to measure the quality of the network output results. The loss function consists of the loss of the contrastive learning module ($L_{UI}$, $L_{TS}^{i}$, $L_{SIS}$) and the loss of the multitasking ($L_{I}$, $L_{S}$), and the different structures of the loss function are connected by the hyperparameter λ.

1. Loss of intent detection task:

(10) $$L_{I}\;≜\;-\sum_{i=1}^{n_{I}}{\hat{y}}_{i}^{I}\log\left(o_{i}^{I}\right)$$

2. Loss of slot filling task:

(11) $$L_{S}\;≜\;-\sum_{j=1}^{n}\sum_{i=1}^{n_{S}}{\hat{y}}_{j}^{i,S}\log\left(o_{j}^{i,S}\right)$$

The total loss function is a linear combination of the losses from the three comparison learning modules and the two tasks:

(12) $$L=\lambda_{I}L_{I}+\lambda_{S}L_{S}+\lambda_{LI}L_{LI}+\lambda_{LS}L_{LS}+\lambda_{GIS}L_{GIS}$$

## 4. Experimental Setup

### 4.1. Experimental Data

In this paper, we conduct experiments on the public datasets MultiATIS++ and the in-vehicle dialogue datasets (MIvD) collected by our own constructed in-vehicle dialogue platform in the vehicle domain. MultiATIS++ contains nine languages, including English (en), Spanish (es), Portuguese (pt), German (de), French (fr), Chinese (zh), Japanese (ja), Hindi (hi), and Turkish (tr). The detailed item information corresponding to the datasets is given in Table 1.

MIvD is collected by the onboard device and then manually redacted and annotated. It includes annotations of intent and semantic slots in four main languages, namely, English (en), Chinese (zh), Japanese (ja), and Arabic (ar). The details are summarized in the following Table 2.

To describe the dataset in more detail, this paper is carried out in Table 3 to show some features of the dataset. Utterance is the instruction entered by the user, and increases the robustness of the model; we have not removed the colloquial words. Intent types are the intent labels of the input instructions, and slot values are the ranges of the attributes of the intent labels, and some of the instructions’ slot values are empty.

### 4.2. Evaluation Indicators and Baseline Model

#### 4.2.1. Evaluation Indicators

In this paper, there are three evaluation indicators used in this paper: the Intent Accuracy for the intent detection task, slot F1 for the slot filling task, and the combined indicators overall accuracy for the two tasks. These evaluation indicators are also widely used in other domains of NLU.

- Intent accuracy: Intent Accuracy is used to evaluate the performance of intent detection by calculating the percentage of sentences that correctly predict the intent.
- Slot F1: The performance of slot filling is evaluated using the F1 score, which is the average score of the reconciliation between accuracy and recall. Slot predictions are considered correct when exact matches are found [30].
- Overall accuracy: It calculates the proportion of sentences that correctly predict intents and slots. The indicator takes into account both intent detection and slot filling [13].

#### 4.2.2. Baseline Model

- CoSDA-ML. Qin et al. [12] proposed a dynamic code-switching method for randomly performing multilingual word-level substitutions. For a fair comparison of MIvD, this paper uses Chinese training data and code-switching data for fine-tuning.
- Multilingual-ZeroShot. Train a joint intent detection and slot filling model using English and generalize to other languages by Jitin [31].
- GL-CLEF is a global–local contrastive learning framework for display alignment proposed by Qin et al. [13].
- LAJ-MCJ is a multilevel label-aware contrastive learning framework for display alignment that was proposed by Liang et al. [32].

### 4.3. Experimental Parameters

In this paper, we use the multilingual mBERT model. The mBERT model has the same architecture and training process as the BERT model, which has 12 transformer layers, 768 hidden states, and 12 attention heads. As shown in Table 4, the optimal hyperparameters are selected by comparing the combination of batch size and learning rate. The probability of replacing a word in the contrastive learning module during training is 0.55. For fine-tuning, the maximum length of the sequence is 128, and the batch size is 32. The optimizer chooses Adam with an initial learning rate of 5 × 10^−6^ and a dropout probability of 0.1. The range of the learning rate is as follows: {2 × 10^−7^, 5 × 10^−7^, 1 × 10^−6^, 2 × 10^−6^, 5 × 10^−6^, 6 × 10^−6^, 5 × 10^−5^, 5 × 10^−4^}; batch size {4, 8, 16, 32}; the size of negative samples chosen from {4, 8, 16, 32}, which are the weight parameters of sentence-level intent CL modules, word-level slot CL modules, and semantic-level intent-slot CL modules, respectively.

Hardware labs are equipped with consumer-grade RTX 3090, enterprise-grade A40 general-purpose AI servers, and enterprise-grade high-performance AI training server A100, with the total GPU computing power FP64 ≥ 200 TFlops and FP32 ≥ 3.0 PFlops. The software uses Linux-based miniconda3 with pytorch1.12.1 + python3.7.

## 5. Result Analysis and Discussion

The IABCL framework proposed in this paper has attained superb results on both MultiATIS++ and MIvD datasets, where the results are excellent on the MIvD dataset. To evaluate the effectiveness of the IABCL-based framework, this paper investigates the following three questions.

- RQ1: Does IABCL predict better results than other cross-lingual migration models in the MIvD dataset?
- RQ2: How well does IABCL work on public datasets?
- RQ3: What role do contrastive learning and interactive attention specifically play?

RQ1: Does IABCL predict better results than other cross-lingual migration models in the MIvD dataset?

To verify this problem, this paper conducts a comparative experiment for the IABCL model on MIvD with other basic models, and the results of the experiment are shown in Table 5.

**Figure 3:**
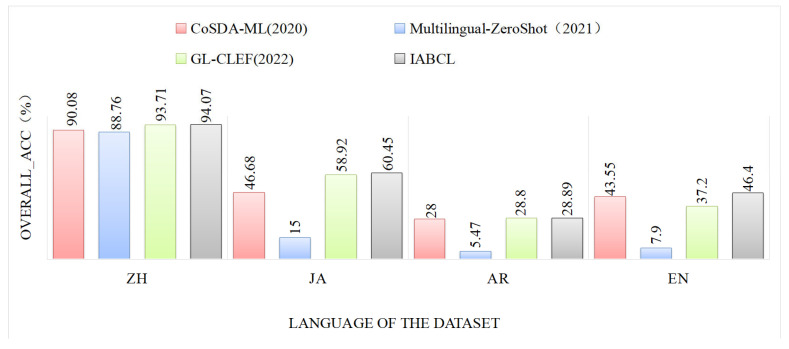
Overall accuracy of MIvD in different languages under baseline.

For the experiments on MIvD, this paper uses mBERT. As shown in Table 5, this paper compares the performance of four different models on different languages and metrics, including intent accuracy, slot F1 value, and overall accuracy. Intent, slot, and overall results have the same trend in different languages. As shown in Figure 3 (results of overall accuracy), IABCL obtained optimal results on MIvD. Compared with other models, this paper improves more significantly in Japanese and English. Because of the cost involved, Multilingual-ZeroShot needs to translate each dataset, and this experiment only translates one-third of the vocabulary comparable with other split-word translations.

Since IABCL and other baseline models are trained based on the Chinese language, they have excellent performance in Chinese onboard datasets; e.g., IABCL achieves 97.40% in Chinese intent accuracy, 95.72% in slot f1 value, and 94.07% in overall accuracy. It is worth noting that in cross-lingual transfer learning, the results of Japanese are better than those of English and Arabic because Chinese and Japanese are of similar language families, and the results of English and Arabic are slightly inferior to those of Japanese, which further verifies that the effect of cross-lingual transfer learning in the same language family is more effective than that of cross-lingual transfer between languages of different language families. Meanwhile, we also analyze the results in English and Arabic separately. The MIvD dataset contains high-dimensional semantic data, such as “I am sweating”, “Here we go”, and “كثيراً حلوة الأغنية هذه” (This song is very sweet), which requires more complex semantic modeling to understand. Additionally, Arabic is a right-to-left language, and this style also affects the final result. Relative to the three baseline models, the framework proposed in this paper excels in cross-lingual transfer learning ability, comparing CoSDA-ML, Multilingual-ZeroShot, and GL-CLEF, with an average improvement of 5.63%, 25.07%, and 3.27% in intent accuracy in English, Japanese, and Arabic, respectively, and an improvement of 4.59%, 24.63%, 2.24%, and 3.27% in slot F1 values, respectively, and 24.63%, 2.24%, and 5.83%, 35.79%, and 0.54% in overall accuracy, respectively.

Overall, the adoption of the IABCL framework has yielded excellent results in both intent and slot prediction in the field of in-vehicle dialogue system.

RQ2: How well does IABCL work on public datasets?

In this paper, the IABCL framework is validated on the public datasets MultiATIS++, and to validate the impact of different multilingual pretraining models on the performance of this model, two pretraining models, mBERT and XLM-R, are used for comparison, and the experimental results are shown in Table 6. The IABCL framework with mBERT achieves the optimal intent accuracy compared with other models, and the F1 value and overall accuracy are slightly worse, while the IABCL framework with XLM-R achieves the optimal slot F1 and overall accuracy, and is slightly worse in intent accuracy, but also achieves more than 90% in general.

**Figure 4:**
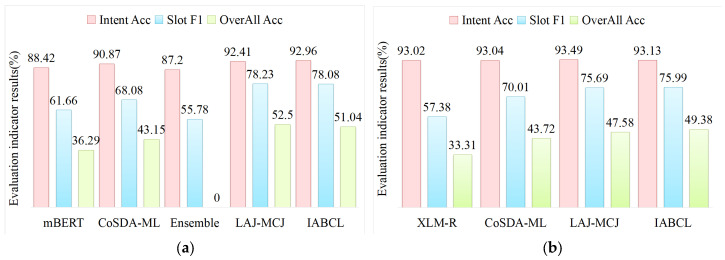
Contrastive learning-based encoder. (**a**) Experimental results of different baseline models under mBERT. (**b**) Experimental results of different baseline models under XLM-R.

As shown in Figure 4, comparing mBERT (2019) and XLM-R (2020) models, this paper improves by 16.42%, 14.75% and 18.61%, 16.01% in slot F1 and overall accuracy, respectively, which verifies that code switch improves significantly on cross-lingual migration. Compared with CoSDA-ML, this paper improves by 1%, 7%, and 6% in intent, slot, and overall, verifying that contrastive learning works significantly on cross-lingual transfer learning. Compared with LAJ-MCJ, this paper only improves the result of intent on mBERT, and the slot and overall are reduced. According to our analysis, in the generation of positive and negative samples of contrastive learning, to reduce the cost, this paper simply takes other utterances from the same batch as negative samples, which may result in the existence of “false-negative samples”, and the final accuracy will be reduced; at the same time, in XLM-R, the accuracy is reduced by 1%, 7%, and 6%. This may cause the existence of “false-negative samples”, and the final accuracy will be reduced; at the same time, in the XLM-R model, the results of slots and overall have been improved, and the intent has been reduced, which verifies that the existence of the “false-negative samples“ will affect the final results.

Taken together, the IABCL model performs better in cross-lingual intent detection and slot filling tasks, and the performance of the IABCL model needs to be improved in Arabic and English. Future research can focus on improving the model’s ability to transfer across language families and exploring more effective methods to achieve cross-lingual transfer learning.

RQ3: What are the specific roles of contrastive learning and interactive attention?

To answer the question, in this paper, we remove the proposed method modules one by one and perform ablation experiments in the MIvD dataset. The results are shown in Table 7. In the table, our-cl is the removal of the contrast learning module, our-bi_interaction is the removal of the attention interaction module, our-all is the removal of both contrast learning and attention interaction modules, and our is the complete model architecture.

Comparing our-all and our-cl (i.e., containing only interactive attention) improves the results in intent, slot, and overall, so the joint framework enables the model to learn more implicit information. Additionally, comparing our and our-cl improves the effect in Sino-Tibetan languages (ZH, JA) more than our-bi_interaction, and we conclude that interactive attention can better act on cognate languages. Further, according to our-bi_interaction, our-all, and our, the boosting effect of contrastive learning in non-Chinese-Tibetan languages is obvious, and contrastive learning can promote cross-linguistic learning of the model and improve the compatibility of the model.

Therefore, in summary, both interactive attention and contrastive learning have their advantages, and the combination of the two is more instructive and practicable.

## 6. Conclusions

To improve the cross-lingual intent detection and slot filling ability of the in-vehicle dialogue system, this paper proposes an interactive attention-based contrastive learning framework (IABCL). Meanwhile, for the first time, this paper constructs multilingual datasets (MIvD) in the field of in-vehicle dialogue, then uses the IABCL framework to verify it, and finally yielded superior outcomes. The experimental results show that the transfer learning can also be centered on the Chinese language and transfer to other languages to obtain a comparable effect.

However, the current results in English and Arabic need to be improved, and we have found that there is a certain correlation between different languages of the same language family, and that the universality of cross-language transfer learning for one or more languages needs to be improved. In the future, based on the Chinese language family, we will further improve the cross-language transfer learning ability of the proposed method to improve the generalization ability of the model.

## Figures and Tables

**Table 1:** MultiATIS++ dataset.

Language	Utterances			Intent Types	Slot Types
	Train	Dev	Test		
en	4488	490	893	18	84
es	4488	490	893	18	84
pt	4488	490	893	18	84
de	4488	490	893	18	84
fr	4488	490	893	18	84
zh	4488	490	893	18	84
ja	4488	490	893	18	84
hi	1440	160	893	17	75
tr	578	60	715	17	71

**Table 2:** MIvD dataset.

Language	Utterances			Intent Number	Slot Number
	Train	Dev	Test		
zh	22,152	2770	2769	17	14
ja	-	392	392	17	14
en	-	501	500	17	14
ar	-	493	493	17	14

**Table 3:** MIvD dataset description.

Language	Utterance	Intent Types	Slot Values
zh	哎帮我把空调调一下19度	adjust_ac_temperture	19
ja	風量を九に	adjust_ac_windspeed	九
en	Open ac cooling mode	open_ac_mode	cooling
ar	درجة ثلاثين على الحرارة درجة تعيين.	adjust_ac_temperature	ثلاثين

**Table 4:** Experiment-related parameters.

Experimental Modules and Parameters	Parameters and Module-Specific Information
Pretrained models	mBERT (12 transformer layers, 768 hidden layers, and 12 attention heads)
Batch size	32
Learning rate	5 × 10^−6^
Probability of code switching	0.55
Maximum sequence length	128
Optimizer	Adam
Dropout	0.1
Number of negative samples	16
λ_1_	0.01
λ_2_	0.005
λ_3_	0.01

**Table 5:** Performance comparison on MIvD.

Intent Accuracy (%)	ZH	JA	AR	EN	Avg
CoSDA-ML (2020)	96.24	60.71	60.32	60.33	69.40
Multilingual-ZeroShot (2021)	96.78	61.22	30.22	31.60	54.95
GL-CLEF (2022)	97.32	72.19	61.86	54.4	71.44
IABCL	97.40	73.97	62.67	61.60	73.86
**Slot F1 (%)**	**ZH**	**JA**	**AR**	**EN**	**avg**
CoSDA-ML (2020)	93.00	72.27	26.77	53.54	61.39
Multilingual-ZeroShot (2021)	90.42	36.52	19.47	36.48	45.72
GL-CLEF (2022)	95.53	75.54	28.62	55.48	63.79
IABCL	95.72	79.68	30.54	56.15	65.22
**Overall Acc (%)**	**ZH**	**JA**	**AR**	**EN**	**avg**
CoSDA-ML (2020)	90.08	46.68	28.00	43.55	52.07
Multilingual-ZeroShot (2021)	88.76	15.00	5.47	7.90	29.28
GL-CLEF (2022)	93.71	58.92	28.8	37.20	54.66
IABCL	94.07	60.45	28.89	46.4	57.33

**Table 6:** Performance comparison on MultiATIS++. Results with * are from LAJ-MCJ.

Method	mBERT			XLM-R		
	Intent Acc (%)	Slot F1 (%)	Overall Acc (%)	Intent Acc (%)	Slot F1 (%)	Overall Acc (%)
mBERT (2019) *	88.42	61.66	36.29	-	-	-
XLM-R (2020) *	-	-	-	93.02	57.38	33.31
CoSDA-ML (2020)	90.87	68.08	43.15	93.04	70.01	43.72
Ensemble-Net (2021) *	87.20	55.78	-	-	-	-
LAJ-MCJ (2022)	92.41	78.23	52.50	93.49	75.69	47.58
IABCL	92.96	78.08	51.04	93.13	75.99	49.38

**Table 7:** Ablation experiments.

Intent Acc (%)	ZH	JA	AR	EN	Avg
Our-cl	97.32	73.27	60.98	58.99	72.64
Our-bi_interaction	96.93	73.19	61.86	60.00	72.99
Our-all	95.66	72.95	59.22	57.80	71.40
Our	97.40	73.97	62.67	61.60	73.91
**Slot F1 (%)**	**ZH**	**JA**	**AR**	**EN**	**Avg**
Our-cl	95.53	76.82	29.84	55.00	62.29
Our-bi_interaction	95.35	75.54	29.92	55.48	64.07
Our-all	93.63	75.30	28.62	54.17	62.93
Our	95.72	79.68	30.54	56.15	65.52
**Overall Acc (%)**	**ZH**	**JA**	**AR**	**EN**	**Avg**
Our-cl	93.71	59.82	28.80	41.40	55.93
Our-bi_interaction	93.64	59.18	28.89	43.80	56.37
Our-all	92.66	55.95	27.18	39.80	53.89
Our	94.07	60.45	30.22	46.40	57.78

## Data Availability

No new data were created or analyzed in this study. Data sharing does not apply to this article.
